# Detection of early stage changes associated with adipogenesis using Raman spectroscopy under aseptic conditions

**DOI:** 10.1002/cyto.a.22777

**Published:** 2015-10-06

**Authors:** Adam Mitchell, Lorna Ashton, Xuebin B. Yang, Royston Goodacre, Alistair Smith, Jennifer Kirkham

**Affiliations:** ^1^ Department of Oral BiologySchool of Dentistry, University of LeedsLeedsUnited Kingdom; ^2^ Department of ChemistryLancaster University, Faraday BuildingLancasterUnited Kingdom; ^3^ School of Chemistry and Manchester Institute of BiotechnologyUniversity of ManchesterManchesterUnited Kingdom; ^4^ Avacta Group Plc., Thorpe Arch EstateWetherbyUnited Kingdom

**Keywords:** adipogenic differentiation, adipose derived stem cell, cell‐based therapy, non‐invasive characterization, principal component analysis, Raman spectroscopy

## Abstract

There is growing interest in the development of methods capable of non‐invasive characterization of stem cells prior to their use in cell‐based therapies. Raman spectroscopy has previously been used to detect biochemical changes commensurate with the osteogenic, cardiogenic, and neurogenic differentiation of stem cells. The aim of this study was to characterize the adipogenic differentiation of live adipose derived stem cells (ASCs) under aseptic conditions. ASCs were cultured in adipogenic or basal culture medium for 14 days in customized culture flasks containing quartz windows. Raman spectra were acquired every 3 days. Principal component analysis (PCA) was used to identify spectral changes in the cultures over time. Adipogenic differentiation was confirmed using quantitative reverse transcription polymerase chain reaction for the marker genes *PPARγ* and *ADIPOQ* and Oil red O staining performed. PCA demonstrated that lipid associated spectral features varied throughout ASC differentiation with the earliest detection of the lipid associated peak at 1,438 cm^−1^ after 3 days of induction. After 7 days of culture there were clear differences between the spectra acquired from ASCs in adipogenic or basal culture medium. No changes were observed in the spectra acquired from undifferentiated ASCs. Significant up‐regulation in the expression of both *PPARγ* and *ADIPOQ* genes (*P* < 0.001) was observed after 14 days of differentiation as was prominent Oil red O staining. However, the Raman sampling process resulted in weaker gene expression compared with ASCs that had not undergone Raman analysis. This study demonstrated that Raman spectroscopy can be used to detect biochemical changes associated with adipogenic differentiation in a non‐invasive and aseptic manner and that this can be achieved as early as three days into the differentiation process. © 2015 The Authors. Published by Wiley Periodicals, Inc. on behalf of the International Society for Advancement of Cytometry.

## INTRODUCTION

Cell‐based therapies are of increasing interest in addressing specific clinical challenges in regenerative medicine as evidenced by the number of ongoing clinical trials 1, 2. The intrinsic properties of stem cells for self‐renewal and differentiation are central to their role in these strategies. However, many of the methods currently employed in the characterization of stem cells and their differentiated progeny are necessarily either invasive or destructive. A minimally invasive methodology for cell characterization that permitted the use of stem cells in regenerative therapies after its application would be advantageous, saving time, money, and resources. Raman spectroscopy is one such non‐invasive method. Raman spectra provide biochemical fingerprints of a sample based on the presence and frequency of the molecular bonds contained within. There are several reported studies where Raman spectroscopy has been used to characterize osteogenic 3, 4, cardiac 5, 6, and neural 7 differentiation of stem cells. However, the majority of studies have been carried out without maintaining culture sterility, thereby rendering the cells unusable for further applications. In the present study we have developed an aseptic methodology to characterize the adipogenic differentiation of adipose derived stem cells (ASCs).

Adipogenic differentiation is one of the distinctive lineages along which mesenchymal stem cells (MSCs), including ASCS, differentiate 8 and is accompanied by a series of well‐defined biochemical/intracellular events. During the initial stages of adipogenic differentiation, stem cells undergo a period of growth arrest and mitotic clonal expansion which triggers the expression of two key pro‐adipogenic transcription factors: peroxisome proliferator‐activated receptor γ (PPARγ) and CCAAT/enhancer binding protein α (C/EBPα) 9, 10, 11. The induction of PPARγ and C/EBPα and subsequent maintenance of their expression is required to produce a mature adipocyte phenotype leading to the expression of several marker genes including fatty acid synthase, fatty acid binding protein, leptin, and adiponectin 12, 13. The concerted action of these genes results in increased glucose uptake, fat accumulation, and insulin sensitivity, producing the characteristic adipocyte histological phenotype of large spherical cells containing many lipid‐rich vacuoles. This is, therefore, a longitudinal process with clear chemical changes; our hypothesis is that Raman spectroscopy should be well suited to its characterization as it can be used to serially analyze the same cellular system without destroying it.

To date there is very little spectroscopic data on the adipogenic differentiation of stem cells reported in the literature, though variants of Raman spectroscopy, surface enhanced Raman spectroscopy (SERS), and coherent anti‐stokes Raman spectroscopy (CARS) have been used 14, 15, 16. Using the SERS approach Moody et al. 14 found that two regions in the Raman spectra best described adipogenic differentiation, 1,200–1,275 cm^−1^ and 1,390–1,610 cm^−1^. Using the CARS approach Mouras et al. 15 found that a peak at 2,845 cm^−1^ could be used to characterize the adipogenic differentiation of ASCs. However, due to limitations in the CARS methodology, namely that excitation can only be supplied for a single vibrational frequency at a time, this was the only molecular species interrogated. In this study, adipogenic differentiation was induced in ASCs over a 14‐day period taking repeated spectra of the same cultures at regular time intervals. The aim was to ascertain if there were any features in the spectra that could indicate differentiation at its earliest stages, before the presence of fat droplets became evident by eye.

## MATERIALS AND METHODS

### Analysis of ASC Adipogenic Differentiation Using Raman Spectroscopy

#### Cell culture

ASCs were purchased from Zen‐Bio (Zen‐Bio, Research Triangle Park, CA). Each batch was comprised of cells from multiple donors: batch 1–6 donors, average age 51, batch 2–8 donors average age 44, batch 3–7 donors, average age 44, batch 4–7 donors, average age 32, and batch 5–9 donors average age 48. These ASCs had tested positive for tri‐lineage differentiation and the cell surface markers CD44 and CD105 and negative for the cell surface markers CD31 and CD45 prior to purchase. Informed ethical consent was obtained by Zen‐Bio prior to isolation and sale. ASCs were supplied at passage 2 and were expanded through 1 further passage by plating 5 × 10^5^ cells in a 75 cm^2^ cell culture flask with Zen‐Bio pre‐adipocyte medium [DMEM/Ham's F‐12 (1:1, v/v), HEPES pH 7.4, fetal bovine serum, penicillin, streptomycin, amphotericin B (proprietary medium, concentrations not supplied)] until 80% confluence, then trypsinized. Customized cell culture flasks with quartz windows, suitable for Raman spectroscopy, were prepared by drilling 1‐cm diameter holes in 25 cm^2^ cell culture flasks. Flasks were washed several times with sterile pure water to remove debris and finally sterilized with 70% ethanol. Heat sterilized quartz discs (Global Optics, Bournemouth, UK), 14 mm diameter × 0.15 mm thickness, were glued to the outside of the flasks using a ultraviolet (UV) cured methacrylate glue (Loctite, Hempstead, UK) and cured for 5 min under UV light. To ensure complete sterilization, flasks were placed under a UV lamp for a further 90 min and periodically rotated to ensure penetration to all surfaces. ASCs suspended in pre‐adipocyte medium were pipetted directly on to the quartz surfaces at 4 × 10^4^ cells/cm^2^. ASCs were left for 48 hours to adhere then either adipo‐induced using adipogenic induction medium supplied by Zen‐Bio [DMEM/Ham's F‐12 (1:1, v/v), HEPES pH 7.4, fetal bovine serum, biotin, pantothenate, human insulin, dexamethasone, isobutylmethylxanthine, PPARγ agonist, penicillin, streptomycin, amphotericin B (proprietary medium, concentrations not supplied)] or cultured in pre‐adipocyte medium. In both cases the flasks were incubated at 37°C under 5% CO_2_. This time point was designated as day 0. As directed by Zen‐Bio, at Day 7 half the adipogenic induction medium was aspirated and replaced with adipogenic maintenance medium, also supplied by Zen‐Bio [DMEM/Ham's F‐12 (1:1, v/v), HEPES pH 7.4, fetal bovine serum, Biotin, pantothenate, human insulin, dexamethasone, penicillin, streptomycin, amphotericin B (proprietary medium, concentrations not supplied)]. To assess the effects of Raman spectroscopy and the Raman sampling process itself on ASCs, an equal number of cultures were maintained in adipogenic or pre‐adipocyte medium and treated in exactly the same way but did not undergo Raman analysis. Each individual batch of ASCs was analyzed once. At no point during the cell culture period was there any evidence of bacterial or fungal contamination.

#### Raman spectroscopy

Raman spectroscopy was performed using a Renishaw RM series 1000 spectrometer with a Leica DMLM microscope attached. Prior to analysis, the cell culture medium was aspirated and flasks filled with Hanks balanced salt solution (HBSS) with 0.45% added glucose. Spectra were acquired using a 40×/0.8 NA water immersion objective immersed in an aliquot of HBSS on the external quartz surface with the flask inverted toward the objective. Spectra were acquired throughout the differentiation period at days 0, 3, 7, 10, and 14 (*n* = 15 for each batch sample, *n* = 75 at each time point, each from a different random locus/cell). Spectra were acquired over the 600–1,800 cm^−1^ wavenumber range with a 120 second exposure from a 785 nm laser providing approximately 66 mW of power on the sample. Cell free areas of quartz were used to acquire background spectra under the same parameters. After analysis the HBSS was aspirated and replaced with the appropriate medium.

#### Data analysis

Pre‐processing of spectra was performed in Grams/32 (Thermo Scientific, Waltham) with further processing and validation by principal component analysis (PCA) performed in Matlab version R2013b (Mathworks, MA). In Grams each individual spectrum had the quartz background subtracted and was smoothed using a seven point Savitzky–Golay filter. All spectra were then exported to Microsoft Excel. An average of 5 spectra, one from each ASC batch, was calculated for every time point and exported to Matlab. Second derivative spectra were calculated and further smoothing performed. PCA was then carried out to visualize the fully processed data.

### Adipogenic Marker Gene Expression Determined by qRT‐PCR

RNA was isolated and reverse transcribed as follows. Undifferentiated ASCs and basal and adipo‐induced ASCs following 14 days in culture were lysed in Trizol^®^ (Invitrogen, Paisley, UK) for 20 min at room temperature. ASCs that had not undergone Raman analysis but were cultured in parallel were also lysed. RNA was extracted from cell lysates using an RNeasy mini kit with additional DNase digestion (Qiagen, Crawley, UK) following the manufacturers recommendations. RNA concentrations were quantified using a NanoDrop spectrophotometer (Thermo Scientific, Wilmington). A High Capacity RNA‐to‐cDNA Kit (Applied Biosystems, Carlsbad) was used to prepare 200 ng of cDNA in 20 µL reaction volumes as per the manufacturer's instructions. Reactions were run on a MJ Research PTC‐100 Thermo Cycler, at 37°C for 1 hour and 95°C for 5 min. Quantitative reverse transcription polymerase chain reaction (qRT‐PCR) was performed using TaqMan gene expression assays (Applied Biosystems, Carlsbad,) for peroxisome proliferator‐activated receptor gamma (*PPARγ*) and adiponectin (*ADIPOQ*): assay numbers Hs01115513_m1 and Hs00605917_m1, respectively, to determine adipogenic marker gene expression. Optimal cDNA concentration was determined as 5 ng/reaction by plotting standard curves with known cDNA concentrations. Data were normalized to tyrosine 3‐monooxygenase/tryptophan 5‐monooxygenase activation protein, zeta (*YWHAZ*) assay number Hs00237047_m1, and analyzed using the comparative cycle threshold method (ΔCT). Graphs of the mean ± SE were plotted in Microsoft Excel and statistical significance was determined by one‐way ANOVA using GraphPad Instat 3 software.

### Lipid Accumulation Determined by Oil Red O Staining

To test for the presence of lipid droplet accumulation within the ASCs following their differentiation, Oil red O staining was performed on adipo‐induced and basal cultured samples ± sampling by Raman spectroscopy after 14 days. Prior to staining, a 0.5% stock solution of Oil red O was prepared in 100% isopropanol (Sigma‐Aldrich, Gillingham, UK). A working solution was created by diluting the stock 3:2 in de‐ionized water and filtered prior to use. ASCs were first washed with PBS then fixed in NBF for 1 hour at room temperature. ASCs were then washed with de‐ionized water, followed by 60% isopropanol and then stained with Oil red O solution for 15 min at room temperature. Excess Oil red O stain was washed away with 60% isopropanol. Finally, to stain cell nuclei, samples were stained with Hematoxylin (Sigma‐Aldrich, Gillingham, UK) for 30 seconds and the excess stain washed away with de‐ionized water. ASCs were imaged on a Leica DMI6000b microscope (Leica Microsystems, Milton Keynes, UK).

## RESULTS

### Adipogenic Differentiation of ASCs Characterized by Raman Spectroscopy

**Figure 1:**
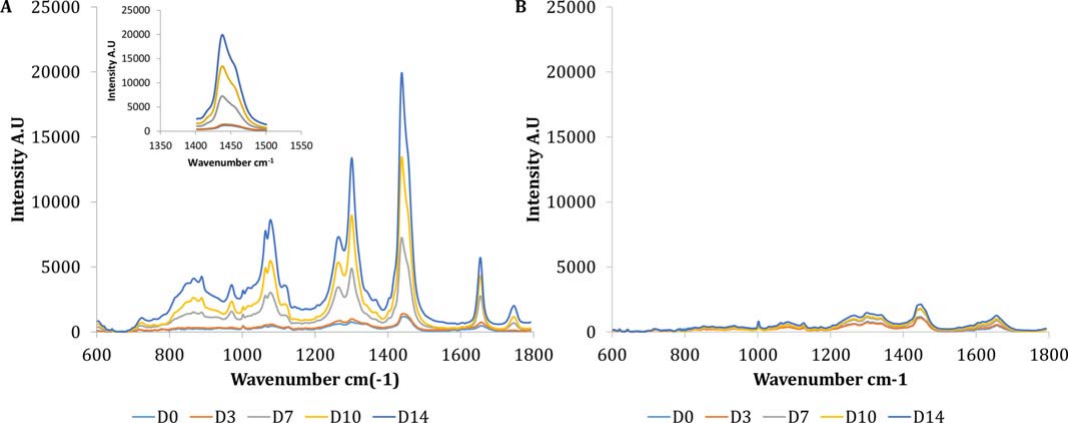
Average background subtracted spectra from adipo‐induced (**A**) and basal cultured (**B**) ASCs taken at days 0, 3, 7, 10, and 14, *n* = 75 spectra per time point from five multidonor batches. Graphs are plotted with matching axes for comparison. A pronounced change in spectra from adipo‐induced ASCs can be seen from Day 7 onward whilst spectra from basal cultured ASCs varied little. Inset is the wavenumber region 1,400–1,500 cm^−1^, the shoulder on the latter portion of the peak indicates the likely presence of two distinct molecular species in this range. [Color figure can be viewed in the online issue which is available at wileyonlinelibrary.com]

The adipogenic differentiation process has rarely been investigated using Raman spectroscopy 14, 15 but we believe that it is potentially well suited to such analysis due to the prominent biochemical changes associated with it. Figure 1 depicts the average spectra for each time point (days 0, 3, 7, 10, and 14 of culture) for ASCs cultured under adipogenic conditions or in basal culture. There was a clear increase in the intensity of several peaks, including the characteristic CH_3_, CH_2_ group in lipids at 1,302 cm^−1^ and C=C in lipids at 1,654 cm^−1^ in spectra from adipo‐induced ASCs after 7 days of induction compared with similar spectra obtained from cells in basal culture. There was also a clear increase in intensity of the peaks in the region of 1,400–1,500 cm^−1^ (see inset in Fig. 1), though this could be caused by an increase in the thickness of the cells. The shoulder on the latter portion of this peak indicates the potential presence of two distinct molecular species, CH_2_ in fatty acids at approximately 1,440 cm^−1^ and CH_3_, CH_2_ in collagen at approximately 1,452 cm^−1^ 17.

**Figure 2:**
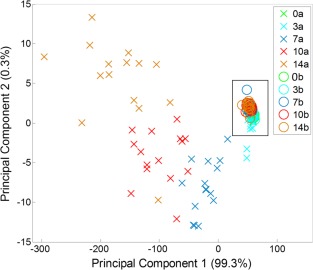
Principal component analysis of second derivative spectra acquired from (**a**) adipo‐induced and (**b**) basal cultured ASCs at days 0, 3, 7, 10, and 14 of culture. Each point is the average of 5 spectra, one from each multidonor batch. *Boxed* are all the spectra from the basal cultured ASCs, this tight grouping indicates there is little variation between these spectra. Trends can be observed in the spectra from adipoinduced ASCs in both PC1 and PC2 with increasing time in culture as demonstrated by the clustering of spectra within a given time point and away from the spectra acquired under basal culture conditions. [Color figure can be viewed in the online issue which is available at wileyonlinelibrary.com]

PCA was performed on spectra obtained from five different multidonor batches of ASCs. A total of 15 spectra were acquired from each ASC batch at each time point for a total of 75 spectra at each time point, data was then averaged using one spectrum from each ASC batch per time point which were then used for PCA. Figure 2 is a two‐dimensional (2D) scatter plot of PC1 and PC2 scores accounting for 99.3% and 0.3% of the variation in the data set, respectively. There was a clustering of all the spectra from ASCs in basal culture and the majority of the spectra at days 0 and 3 from the adipo‐induced ASCs (boxed in the figure). However, spectra from adipo‐induced ASCs at days 7, 10, and 14 clearly cluster away from this and from one another. This resulted in two trends, firstly in PC1 from Day 0 to 14, secondly in PC2 from Day 0 to 7 and then Day 7 to 14.

**Figure 3:**
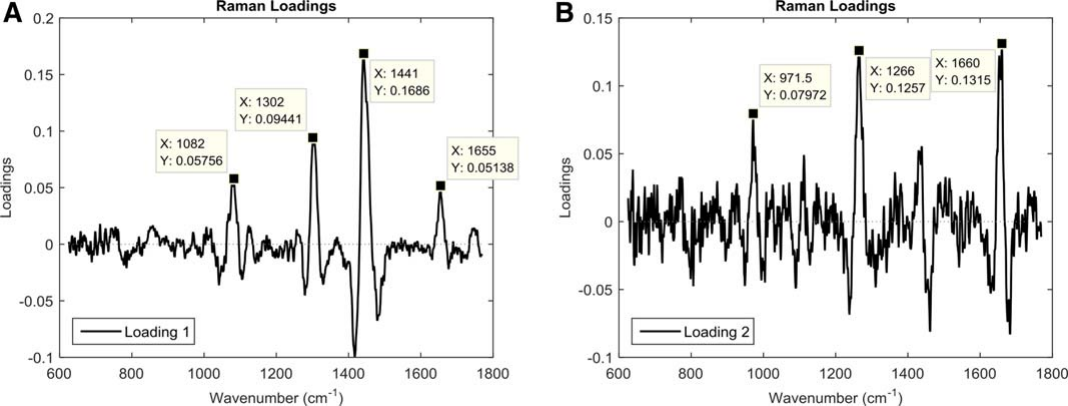
The individual loadings for PC1 (**A**) and PC2 (**B**) from the PCA of ASCs in adipogenic and basal culture. Significant peaks of interest are indicated where X provides the wavenumber of the molecular vibration. This includes molecular vibrations associated with lipids (1,082, 1,302, and 1,441 cm^−1^) and proteins (971.5, 1,266, and 1,660 cm^−1^).

In order to ascertain which peaks were responsible for the variation within the principal components, the loadings for each PC were plotted (Figs. 3A and 3B). The loadings for PC1 showed peaks for wavenumbers corresponding to C—N bonds in proteins and lipids (1,082 cm^−1^), CH_3_, CH_2_ in lipids (1,302 cm^−1^), CH_2_ in fatty acids (1,441 cm^−1^), and C=C bonds in lipids (1,654 cm^−1^). Similarly, the loadings plot for PC2 had peaks corresponding to C—C bonds in collagen (971.5 cm^−1^), and amide bonds (amide III at 1,266 cm^−1^ and amide I at 1,660 cm^−1^). Putative peak assignments were based on Movasaghi et al. 17.

**Figure 4:**
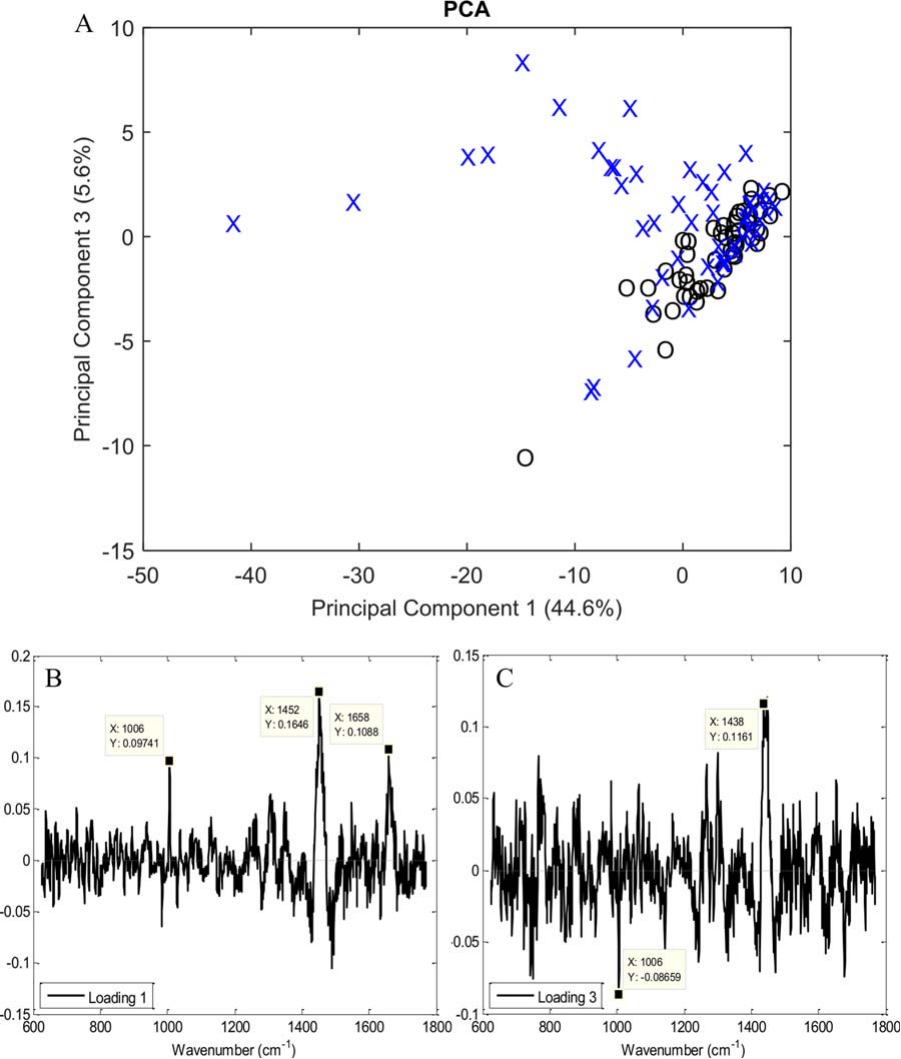
(**A**) Principal component analysis of spectra from (X) adipo‐induced and (O) basal cultured ASCs after 3 days. It can be seen that even at this very early stage of adipogenic culture, spectra from adipoinduced ASCs are beginning to cluster away from spectra obtained from cells in basal medium. (**B and C**) Loadings for PC1 and 3, respectively, from the PCA of ASCs in adipogenic culture and basal medium. Marked are significant peaks of interest where X provides the wavenumber of the molecular vibration.

**Figure 5:**
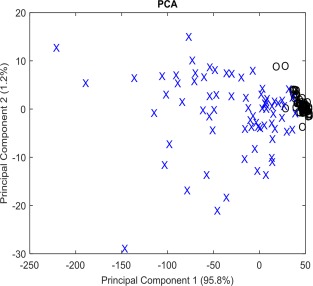
Principal component analysis of spectra from (X) adipo‐induced and (O) basal cultured ASCs after 7 days. It can be seen that after 7 days of adipogenic induction the majority of the spectra acquired from induced ASCs have clustered away from the spectra acquired from ASCs in basal conditions marking the point where the two are likely phenotypically distinct.

In order to determine if any spectral features were indicative of the early differentiation process, PCA was performed on spectra from ASCs cultured in adipogenic or basal medium at Day 3 (Fig. 4A). Principal components 1 and 3 were plotted accounting for 44.6% and 5.6% of the variation, respectively, PC2 was not correlated with any interesting biological feature and so is not shown. All of the spectra from the ASCs cultured in basal medium and a majority of those from the adipo‐induced ASCs were clustered together. However, a small number of spectra (∼10%) were separated from the main cluster based on contributions from both PC1 and PC3 in the adipogenic culture group. The loadings for PC1 and PC3 (Figs. 4B and 4C) further indicated that there were two potential molecular species in the prominent 1,400–1,500 cm^−1^ region, the first in PC1 at 1,452 cm^−1^ and the second in PC3 at 1,438 cm^−1^. As previously suggested these may represent CH_3,_ CH_2_ in collagen, and CH_2_ in fatty acids, respectively. The remaining peaks in PC1 correspond to proteins: phenylalanine (1,006 cm^−1^) and amide I (1,655 cm^−1^). Finally, PCA was performed on spectra from ASCs cultured in adipogenic or basal medium at Day 7 (Fig. 5). Principal components 1 and 2 were plotted accounting for 95.8% and 1.2% of the variation, respectively. At this time point the vast majority (>90%) of the spectra acquired from the ASCs undergoing adipogenic differentiation had separated away from the spectra acquired from ASCs in basal culture. This too was attributable to molecular vibrations associated with lipids. Figures 4 and 5 suggest that the adipogenic differentiation process may be detectable after just 3 days and that the cells undergoing differentiation become phenotypically distinct within 7 days.

### Adipogenic Differentiation of ASCs ± Analysis by Raman Spectroscopy Using qRT‐PCR And Oil Red O Staining

**Figure 6:**
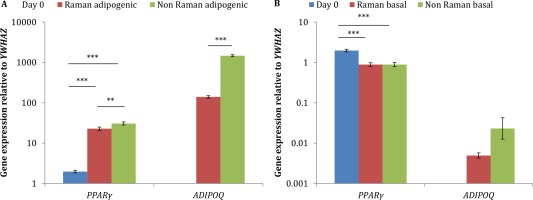
A comparison of ASC adipogenic marker gene expression at Day 0 and Day 14 from ASCs in (**A**) adipogenic and (**B**) basal culture that had/had not undergone Raman spectroscopy. Results show mean ± SE (*n* = 9). ***P < 0.001, **P < 0.01. Statistical significance was determined using a one‐way ANOVA. [Color figure can be viewed in the online issue which is available at wileyonlinelibrary.com]

In order to confirm the adipogenic differentiation of ASCs the expression of two adipogenic marker genes *PPARγ* and *ADIPOQ* were measured and Oil red O staining used to detect lipid droplet accumulation. In addition, both measurements were used on control cells and those exposed to the laser used for Raman spectroscopy (785 nm with ∼66 mW on the cells) in order to determine if the Raman acquisition process had had any effect on adipogenesis. Significant increases in *PPARγ* and *ADIPOQ* expression were observed during adipogenic induction irrespective of the Raman acquisition process (*P* < 0.001 for *PPARγ*, *ADIPOQ* was undetectable at Day 0) (Fig. 6A). However, there was significantly less expression of *PPARγ* and *ADIPOQ* in ASCs that had been subjected to Raman analysis compared with those that had not been analyzed by Raman (*P* < 0.01 and *P* < 0.001, respectively). The expression of both marker genes was also analyzed in ASCs cultured in basal medium (Fig. 6B). A significant decrease in the expression of *PPARγ* was observed after 14 days irrespective of the Raman acquisition process (*P* < 0.001). However, no significant differences in the expression of either gene were observed between ASCs that had/had not undergone Raman analysis.

**Figure 7:**
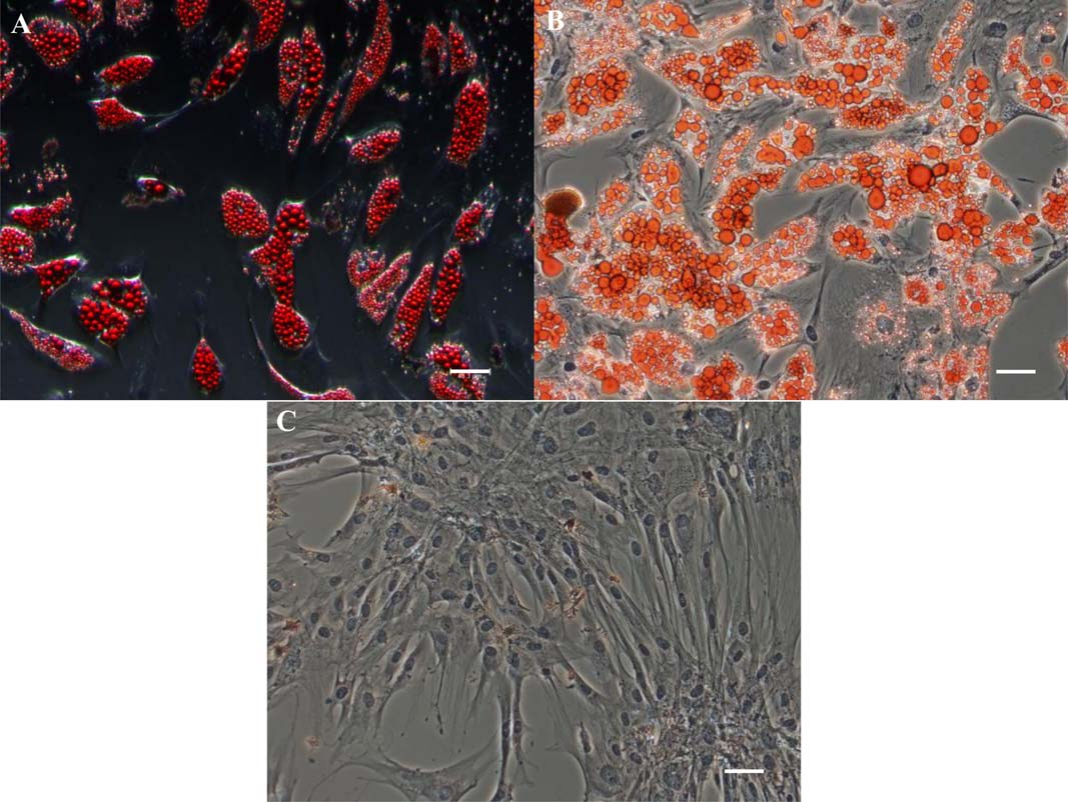
Oil red O staining of ASCs after 14 days of adipo‐induction for cells that (**A**) had undergone Raman measurements, and (**B**) had not undergone Raman measurements, (**C**) Oil red O staining of ASCs after 14 days of culture in basal medium after having undergone Raman measurements. Scale bar = 50 μm. [Color figure can be viewed in the online issue which is available at wileyonlinelibrary.com]

Oil red O staining confirmed the presence of lipid droplets in adipo‐induced ASC cultures after 14 days regardless of whether the cells had undergone Raman analysis or not (Figs. 7A and 7B). No lipid droplets were detected in the basal cultured ASCs (Fig. 7C).

## DISCUSSION

The aim of this study was to determine whether or not it was possible to detect the adipogenic differentiation of ASCs by Raman spectroscopy where a series of Raman spectra were taken from the same cell cultures over time, using an aseptic methodology. Adipogenesis was selected for characterization as spectroscopic data in the literature is sparse for this differentiation lineage and the process lends itself well to analysis by Raman spectroscopy. Two previously reported studies used variants of Raman spectroscopy, surface enhanced Raman spectroscopy (SERS) 14 and coherent anti‐stokes Raman spectroscopy (CARS) 15. SERS cannot be considered non‐invasive as it requires the addition of metal nanoparticles to the cells (which may inadvertently alter cellular metabolism) and CARS generally requires that one preselects the molecular vibration of interest to image which requires a priori knowledge of the markers of interest. For a full description of the principles behind Raman spectroscopy please refer to Downes and Elfick (2010) 18.

In the present study, we used PCA to analyze spectra acquired from ASCs throughout the adipogenic differentiation process during 14 days of adipogenic culture and compared these data with data acquired from ASCs in basal culture. For a description of PCA please refer to Bro and Smilde (19) 19. Under adipogenic conditions, the PCA scores plot for PC1 versus PC2 demonstrated a continuous trend in PC1 from Day 0 to Day 14 and a trend in PC2 from Day 0 to Day 7 followed by a trend from Day 7 to day 14. It is clear that adipogenic differentiation has a very large effect on cellular phenotype as PC1 encompassed the vast majority of the variation in the data set [the total explained variance (TEV) was 99.3%]. When the loadings for PC1 were plotted, it was found that several peaks indicative of lipids and fatty acids were behaving in a similar fashion whilst peaks indicative of nucleic acids were behaving in an opposing manner. PC2, whilst only accounting for 0.3% of the TEV in the data set, comprised of peaks relating to protein content. When the loadings for PC1 and PC2 were plotted against one another, it was demonstrated that lipid associated features were most variable early on in the differentiation process, between days 3 and 7. Features associated with nucleic acid and protein varied most at the end of the process, at day 14. This is broadly in agreement with the study by Moody et al. (14) 14 who also determined that peaks in the region 1,390–1,610 cm^−1^ varied most during the adipogenic differentiation process, many of which were also found to vary in this study. The spectra from ASCs cultured in basal medium varied little over the course of 14 days and these spectra formed a tight cluster upon PCA underlining the lack of variation in these cells in the absence of inductive cues. Finally, to ascertain if any spectral features may have been predictive of the differentiation process, PCA was performed on spectra from ASCs cultured under adipogenic and basal conditions after 3 days of culture. The results indicated that spectra from the adipo‐induced cells were beginning to separate away from spectra obtained from cells cultured in basal medium. The loadings indicated that this was due to a single lipid associated peak at 1,438 cm^−1^. By Day 7 there was a clear separation between the spectra acquired from induced ASCs compared with ASCs in basal culture. This suggests that between 3 and 7 days of adipogenic induction, biochemical changes take place that are indicative of differentiation. While we are unable to determine specificity/sensitivity of this method as a diagnostic for early differentiation using the current data, our results point the way for further studies that could focus on this time period with a view to determining the precise timing of this biochemical change and the spectral features associated with it. Raman spectroscopy may then ultimately be used in a translational setting. Additionally, confirmation of MSC adipogenic differentiation using conventional techniques such as Oil red O staining or qRT‐PCR usually occurs between 7 and 21 days 8, 13, 20, the Raman spectra in this study indicated that adipogenesis can potentially be detected several days earlier and in a non‐invasive manner. qRT‐PCR and histological staining were used to confirm that adipogenic differentiation had indeed occurred over the culture period and to determine if the process had been affected by the Raman sampling method. There was a significant increase in the expression of both *PPAR*γ and *ADIPOQ* marker genes following 14 days of adipogenic induction and Oil red O staining confirmed that adipo‐induced ASCs had produced lipid‐rich vacuoles. A mature adipocyte phenotype was therefore observed in adipo‐induced ASCs irrespective of whether or not they had undergone Raman analysis but the expression levels of adipogenic marker genes was significantly reduced in ASCs that had undergone Raman analysis. This was most prominent in an approximately 10‐fold reduction in the expression of *ADIPOQ*, however, it is difficult to ascertain if this reduction in gene expression was biologically significant as a mature adipocyte phenotype (the presence of lipid‐rich vacuoles) was observed following Oil red O staining. A review by Vogel and Marcotte (21) 21 explored the relationship between mRNA and protein production and reported that only 40% of the variation in protein levels can be attributed to mRNA levels the rest resulting from post‐transcriptional regulation, indicating that some changes in mRNA levels may not necessarily result in altered cellular phenotype. However, it is widely accepted that there is a dysregulation of adipogenesis and the associated marker genes in obesity as a result of hypoxia 22. Indeed, several studies have demonstrated that *ADIPOQ* expression is reduced under either persistent or intermittent hypoxia 23, 24, 25 such as might be anticipated during prolonged Raman sampling using our method.

Raman spectroscopy is a technique capable of producing a biochemical “fingerprint” of a given sample. In the instance of characterizing the differentiation of stem cells; if the differentiation process results in changes to cellular biochemistry over time then Raman spectroscopy is well suited to detecting such changes. For example the osteogenic differentiation process has been shown to be characterized by the emergence of peaks at 960 cm^−1^ and 1,070 cm ^−1^ associated with molecular species found within hydroxyapatite 3, 4. Similarly, during cardiogenic differentiation it has been found that molecular species found within α‐actinin and glycogen (860 cm^−1^ and 938 cm^−1^, amongst others) best describe this process using Raman spectroscopy 5, 6. In the present study, and perhaps unsurprisingly, we have found that molecular species, with peaks at 1,302 cm^−1^ and 1,441 cm^−1^, found within lipids, [the assignments of which are based on the work of others, reviewed in Ref. 17], best characterize the adipogenic differentiation process.

In conclusion this study demonstrates that it is possible to monitor key biochemical changes that occur during the adipogenic differentiation of ASCs, namely the accumulation of intracellular lipids, using Raman spectroscopy. This can be achieved in an entirely non‐invasive way without compromising the sterility of the cell cultures. However, there was evidence that either exposure to Raman spectroscopy or the sampling methodology reduced adipogenic differentiation but not to a degree that prevented it. Refinements to our method such as acquiring spectra using an environmentally contained microscope may ameliorate the observed reduction in gene expression. Our method in addition to such improvements would not only be important in demonstrating that Raman spectroscopy is a viable tool in the monitoring of stem cell differentiation but could also signpost toward an application that is appropriate to the scaled up production of large numbers of quality controlled patient specific stem cells for clinical use.

## Supporting information

Supporting Information

Supporting Information
