# Supplementary figures and images for: Detection of early stage changes associated with adipogenesis using Raman spectroscopy under aseptic conditions
Source: Cytometry A. 2015 Oct 6;87(11):1012–9. doi: 10.1002/cyto.a.22777 (PMC4832334; doi:10.1002/cyto.a.22777)

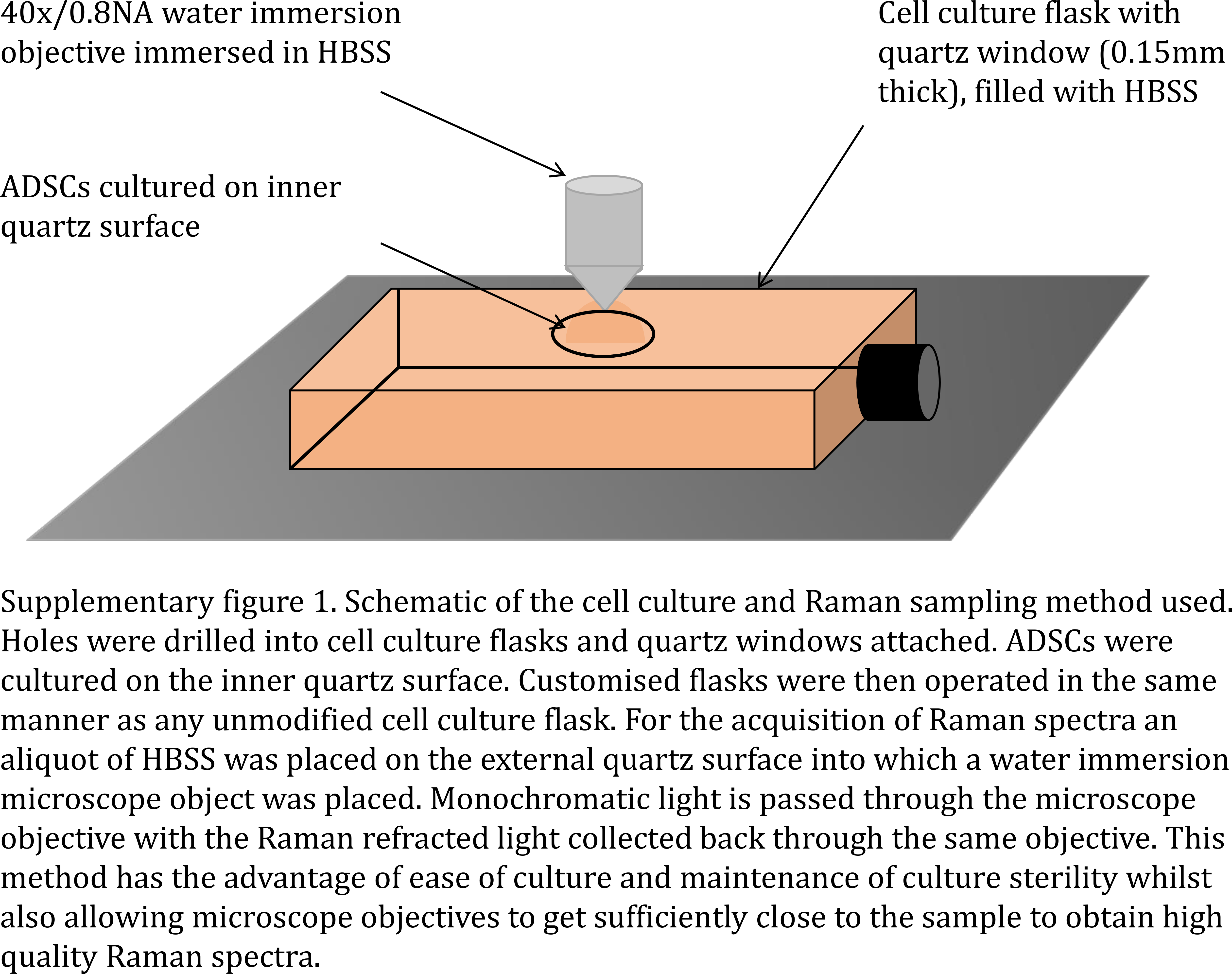

Supplement: Supplementary file 1 — Supporting Information [file CYTO-87-1012-s001.tiff]
