# Supplementary material for: Detection of early stage changes associated with adipogenesis using Raman spectroscopy under aseptic conditions
Source: Cytometry A. 2015 Oct 6;87(11):1012–9. doi: 10.1002/cyto.a.22777 (PMC4832334; doi:10.1002/cyto.a.22777)
Supplement: Supplementary file 2 — Supporting Information [file CYTO-87-1012-s002.docx]

Written in response to:

*Provide an online supplement explaining Raman spectroscopy methods and data analysis in relation to this project (particularly how lipid and nucleic acid signals are inferred) in terms that a flow or image cytometrist can understand.*

In the most basic sense, spectroscopy can be described as the study of matter using electromagnetic radiation ([1](#_ENREF_1)). The electromagnetic (EM) spectrum describes light as a function of its wavelength from sub nanometre gamma rays to radio waves in the order of metres. Raman spectroscopy when used to analyse biological tissues typically uses a light source from the near infra red portion of the spectrum. Other light sources such as ultra violet can be used for Raman spectroscopy, however, light of this wavelength is unsuitable for cellular analysis due to the potential for photo-induced mutagenicity ([2](#_ENREF_2)). Raman spectroscopy measures the difference in frequency between incident and emitted light/photons, termed a Raman shift. The difference is the result of interaction with the molecules of a sample and a change in the vibrational energy state of those molecular bonds, for a detailed description please refer to ([3](#_ENREF_3)). A plot of all the Raman shifts observed in a sample produces a spectrum where shifts are characterised in wavenumbers (inverse of wavelength in cm^-1^) from the incident frequency. The Raman shift of many molecular motifs such as CH_3_ can be calculated on the basis atomic mass and structure amongst other things. Please refer to an excellent study by Shimanouchi *et al* 1978 ([4](#_ENREF_4)) for a detailed description of this calculation for over 100 chemical motifs. In complex biological systems investigators may wish to know more than just the individual molecular vibrations present in their sample such as the identity of the larger molecules from which those molecular vibrations were derived. This can in part be achieved by comparing spectra of pure molecules to those of the sample. Gentleman *et al* 2009 ([5](#_ENREF_5)) used spectra acquired from types I and II collagen and synthetic carbonated hydroxyapatite and used them to infer their presence in the cell samples undergoing osteogenic differentiation. Indeed many molecules have been inferred/identified in a similar manner. In a review, Movasaghi *et al* 2007 ([6](#_ENREF_6)) compiled data from many papers were Raman spectroscopy had been used to study biological tissues. In the papers reviewed peak assignments were made both by consultation with the calculated vibrational frequencies of chemical motifs and the measurement of pure molecular standards. This review and the papers contained within formed the basis for our own putative peak assignments.

Raman spectra typically have many data points most of which represent biochemically important data, all of which have the potential to be associated with one another. As such there is a need for techniques capable of uncovering and describing this inherent complexity. Multivariate analysis techniques achieve this by simplifying a data set and so aiding its visualisation and may also permit classification or prediction of outcome ([7](#_ENREF_7)). One commonly used technique for analysing Raman spectra is principal component analysis (PCA) and was also the technique used for our own analysis. PCA, takes a data matrix (tabulated spectral data) and reduces it into principal components (PCs). PCs describe the variance of the data set where PC1 contains the most variation, PC2 the second most variation and so on. In the case of spectral data, this means that the wavenumbers (peaks that relate to particular molecular species) that vary most are grouped in PC1 and so on. In order to visualise the data, each spectrum in the data matrix is assigned a score and each wavenumber a loading for each PC. Scores and loadings can both be plotted and provide complementary results. A tutorial review of PCA by Bro and Smilde 2014 ([8](#_ENREF_8)) is an excellent beginners guide to PCA and uses simplified examples to demonstrate the method and guide in its analysis. Briefly, in one example they take 44 different wines produced in 4 regions (Argentina, Australia, Chile and South Africa) and compared them on the basis of 14 different variables (ethanol, total acid, volatileA, malic acid, pH, lactic acid, sugar, citric acid, CO_2_, density, folinC, glycerol, methanol and tartaricA). The aim of the analysis was to determine if wines produced in the same region were similar to one another but different to wines from other regions and if this was the case which variables were responsible. When the scores were plotted (as a 2D scatter plot) for PC’s 1 and 2 it indicated that wines from within a region were similar to one another (demonstrated by those wines forming clusters on the plot) and that wines from Chile and Australia were most dissimilar (demonstrated by wines from those regions forming clusters in different sections of the plot). The loadings for each PC (the variables grouped within a given PC) were then used to determine the parameters responsible for the difference between Chilean and Australian wines. This was found to be primarily ethanol and CO_2_ content. We highly recommend this review for any researchers seeking a beginners guide to PCA, when it is best applied and how to interpret the results.

1. Ball DW. The Basics of Spectroscopy. Bellingham: SPIE; 2001.

2. Rasmussen RE, Hammerwilson M, Berns MW. Mutation and sister chromatid exchange induction in Chinese-hamster ovary (CHO) cells by pulsed excimer laser-radiation at 193 nm and 308 nm and continuous UV-radiation at 254 nm. Photochem Photobiol. 1989;49(4):413-8.

3. Andrews DL, and Demidov, A. A. An Introduction to Laser Spectroscopy. New York: Plenum Press; 1995.

4. Shimanouchi T, Matsuura H, Ogawa Y, Harada I. Tables of molecular vibrational frequencies. Journal of Physical and Chemical Reference Data. 1978;7(4):1323-444.

5. Gentleman E, Swain RJ, Evans ND, Boonrungsiman S, Jell G, Ball MD, et al. Comparative materials differences revealed in engineered bone as a function of cell-specific differentiation. Nat Mater. 2009;8(9):763-70.

6. Movasaghi Z, Rehman S, Rehman IU. Raman spectroscopy of biological tissues. Appl Spectrosc Rev. 2007;42(5):493-541.

7. Rajalahti T, Kvalheim OM. Multivariate data analysis in pharmaceutics: A tutorial review. International Journal of Pharmaceutics. 2011;417(1-2):280-90.

8. Bro R, Smilde AK. Principal component analysis. Analytical Methods. 2014;6(9):2812-31.
